# SiNC: Saliency-injected neural codes for representation and efficient retrieval of medical radiographs

**DOI:** 10.1371/journal.pone.0181707

**Published:** 2017-08-03

**Authors:** Jamil Ahmad, Muhammad Sajjad, Irfan Mehmood, Sung Wook Baik

**Affiliations:** 1 College of Software and Convergence Technology, Department of Software, Sejong University, Seoul, Republic of Korea; 2 Digital Image Processing Lab, Department of Computer Science, Islamia College, Peshawar, Pakistan; 3 Department of Computer Science and Engineering, Sejong University, Seoul, Republic of Korea; Northwestern University Feinberg School of Medicine, UNITED STATES

## Abstract

Medical image collections contain a wealth of information which can assist radiologists and medical experts in diagnosis and disease detection for making well-informed decisions. However, this objective can only be realized if efficient access is provided to semantically relevant cases from the ever-growing medical image repositories. In this paper, we present an efficient method for representing medical images by incorporating visual saliency and deep features obtained from a fine-tuned convolutional neural network (CNN) pre-trained on natural images. Saliency detector is employed to automatically identify regions of interest like tumors, fractures, and calcified spots in images prior to feature extraction. Neuronal activation features termed as neural codes from different CNN layers are comprehensively studied to identify most appropriate features for representing radiographs. This study revealed that neural codes from the last fully connected layer of the fine-tuned CNN are found to be the most suitable for representing medical images. The neural codes extracted from the entire image and salient part of the image are fused to obtain the saliency-injected neural codes (SiNC) descriptor which is used for indexing and retrieval. Finally, locality sensitive hashing techniques are applied on the SiNC descriptor to acquire short binary codes for allowing efficient retrieval in large scale image collections. Comprehensive experimental evaluations on the radiology images dataset reveal that the proposed framework achieves high retrieval accuracy and efficiency for scalable image retrieval applications and compares favorably with existing approaches.

## 1. Introduction

Rapid technological advances in medical imaging devices facilitate generation, transmission, consumption, and storage of medical images in hospitals and clinics [1]. The growing dependency on recent medical diagnostic methods like radiology, histopathology, and computed tomography causes massive increase in the volume of digital images stored and processed on a regular basis. These data-stores containing images of different modalities like X-Ray, CT, MRI, ultrasound, and PET have become essential sources of anatomical and functional information which can assist in diagnosis, education, and research [2]. They serve as a vital resource for providing decision support to radiologists and medical experts (ME) through retrieval of relevant medical case records and images from huge medical multimedia collections [3]. These databases grow in volume and diversity with the passage of time, and extraction of relevant information becomes increasingly difficult [4]. Furthermore, the exponential growth in these databases renders manual annotation infeasible. Therefore, content-based image retrieval (CBIR) methods can effectively be used for this purpose.

It is common for MEs to render diagnosis based on experience and intuition [5]. Current picture archival and communication systems (PACS) allow operators in hospitals to store, retrieve, and transmit medical images. However, these systems are based on text-based retrieval techniques which are plagued with many weaknesses [6]. For instance, the inability of text-based approaches to effectively represent images make it difficult to locate relevant images from diverse image collections, thereby compelling physicians to manually browse for their desired contents which is a tiresome practice. These limitations in the text-based PACS systems have led to the development of content-based retrieval methods, where the contents of images are processed for organizing the database. In these methods, access to relevant medical images is carried out at the perceptual level-based on visual features extracted from color, shape, and texture of images, with appropriate image similarity models. CBIR systems are striving to represent medical images semantically, in order to allow timely access to accurate relevant information [7].

At the core of CBIR systems exist an image representation process which attempts to model images using feature vectors of fixed dimensions [8]. A fundamental problem in CBIR systems is to represent images in a manner that their visual as well as semantic similarities can be effectively determined. In typical CBIR systems, feature vectors are constructed by extracting low-level features from color, texture, or shapes to represent images in the database [7]. The proportion of similarities in these feature vectors is used to derive relevance between the query and target images. Images having high visual similarity may be very different in terms of their semantics. This problem is referred to as the *semantic gap*, which reflects the disagreement between low-level features and high level user semantics [9]. In case of image retrieval, the extracted features may fail to represent high level concepts in images which could lead to retrieval of irrelevant images. For instance, a radiologist may wish to look for fractured leg in a collection of radiographs, but the retrieval system may only retrieve normal leg radiographs without any fracture, because most of the image contents are visually similar. Consequently, concepts like machine learning, relevance feedback (RF), adaptive similarity functions, and extraction of semantic features are used to reduce this gap [10].

Regions of interest (ROI) detection is a natural activity in high level perception, where most of the attention is dedicated to processing only a small area of the visual field [11]. These ROIs are areas of images regarded as more vital and meaningful than the rest of the image. The use of ROIs in image description process can effectively improve the representation process by careful consideration of these significant areas [12]. In the context of medical images, areas representing fractured bones or calcified spots are considered more significant than the rest of the image [13]. Recent saliency detection methods can be applied to automatically detect regions of interest in medical images, so that more importance may be given to ROIs than the rest of the image during image description and relevancy computation process. Similarity in anatomical structures, presence of identical medical peculiarity, and visual similarity are the key characteristics for determining content relevancy in medical image retrieval systems [14]. It is essential to consider radiologists’ perspective to build an effective image representation by incorporating visual saliency which the existing systems do not incorporate appropriately. Such a representation depends on the ability of extracted features to model semantics in medical images, thereby making effective image representation the key factor for improving retrieval performance.

Existing content-based medical image retrieval (CBMIR) systems lack the representational capability for radiology images to facilitate efficient retrieval of semantically relevant images from large databases consisting of several terabytes data per year [3]. The lack of color information in radiographs, structural complexity of the human body due to its deformable nature, variation in illumination, and the lack of sufficient information regarding significant ROI in the image description process are some of the reasons for the weaknesses in existing CBMIR systems. In this paper, we propose an effective method to represent medical images by incorporating visual saliency with fine-tuned neural codes to facilitate semantic retrieval of medical images. Furthermore, we provide an efficient method for retrieval in large scale datasets by employing locality sensitive hashing to the proposed SiNC descriptor.

The success of deep CNNs in dramatically improving image classification has motivated the computer vision community to adapt these powerful architectures for other related problems like object detection, image classification, face recognition, and image retrieval [15]. Recent studies have revealed that the features emerging in the higher layers of CNNs contain significant discriminative capabilities for determining image similarities in retrieval applications. Moreover, the activation features from CNNs pre-trained on large datasets such as ImageNet [16] have been successfully employed as generic image representation methods [17, 18]. Significant interests arose after the impressive success of these features in visual recognition, which resulted in several extensions to the work including [19–21]. Interestingly, these features are not only effective at determining visual similarity but also perform exceptionally well at computing semantic consistency in visual contents [22]. Furthermore, efficient and compact representations have also been derived using hashing methods for accessing relevant images in huge image collections and perform other relevant tasks like image segmentation more efficiently [23, 24].

Our major contributions in this work are as follows:

1. Saliency detection is employed to automatically identify ROI involving medical peculiarities like fracture, calcified spots, and tumors in medical images
2. Fine-tuned CNN is used to extract discriminative features (neural codes) from the whole medical image as well as their salient components
3. Neural codes of the salient patch are injected into neural codes of the whole image to represent medical images, allowing their accurate retrieval
4. The weighted fusion scheme for injecting neural codes allow users to adjust the influence of saliency in retrieving images
5. Computation of locality sensitive binary hash codes to enable efficient large scale retrieval

The rest of the paper is organized as: Section 2 presents some of the relevant works regarding the retrieval of radiology images, Section 3 highlights the various aspects of the proposed framework. Experiments and their results are discussed in Section 4. Section 5 concludes the paper with a review on the strengths and weaknesses of our framework along with further research directions.

## 2. Related work

Advancement in the field of CBMIR has benefited medical experts in clinical decision making, medical education, and research. Effective utilization of the visual content resources associated with previous medical cases can provide useful insights and decision support to radiologists and other MEs. To ensure timely availability of such information, researchers are constantly trying to develop efficient medical image retrieval systems. They have developed frameworks to represent medical images using low-level features, bag-of-visual-words (BoVW), and sparse coding-based methods. Seetharaman and Sathiamoorthy [25] proposed a unified learning framework to index and retrieve medical images. Three different low-level features from color and texture including color auto-correlogram [26], edge orientation autocorrelogram [27], and micro-texture information were extracted to represent both color and grayscale medical images. Although color information is missing in case of grayscale images, their work showed that both kinds of images were retrieved with high accuracy. The color auto-correlogram did not capture any color information from such images but captured features among intensity values. For grayscale medical images, besides color and texture, shape features have also been frequently used including Fourier descriptors [28], invariant moments [29], co-occurrence matrices [30], Gabor features [31], and wavelets [32] for representation. The inherent weakness of low-level features in representing high-level concepts (i.e. semantics) limit their ability to accurately retrieve medical images for practical applications [10].

In addition to features extracted directly from color, texture, or shapes, BoVW approaches have been widely used in CBMIR systems. These techniques allow essential features of images to be learned in an unsupervised manner. Local patches from salient keypoints are collected from the pool of training images and clustered to form a codebook of visual words. Each image is then represented as a collection of visual words, usually without any spatial information of these words. The BoVW framework has been extensively studied and improved over the course of years in the domain of medical images. Iakovidis et al. [33] presented a pattern similarity scheme for retrieving medical images and termed their approach as PANDA (Patterns for Next generation DAtabase systems). Their framework was based on the BoVW approach, where low-level features were extracted from small image patches and clustered to form representative patterns or code words. Furthermore, several simple patterns corresponding to a particular anatomical specimen were grouped to form complex patterns. This grouping to construct complex representative patterns effectively adds semantic meaning to these patterns. The number of clusters were determined automatically using expectation maximization approach. The presence of these semantic patterns enabled them to effectively compute similarity between image pairs. In another extension to the BoVW approach, Wang et al. [34] developed a weighted scheme for visual words obtained from dense sampling of radiology images. Weights in their scheme indicate significance of visual words in the image representation. These weights are learned by the adaboost algorithm. Avni et al. [35] introduced a patch-based visual words framework. They applied the BoVW approach to densely sampled rectangular patches to allow organ level and pathological level categorization and retrieval. Their system was able to discriminate between healthy and pathological cases in chest radiographs. In another similar approach, Yang et al [36] used Scale Invariant Feature Transform (SIFT) [37] based interest points to build a BoVW-based representation for medical images. The use of salient patches in representing images allowed their framework to retrieve CT images with similar lesions. The success of BoVW in these works is attributed to the inclusion of saliency information determined by the salient keypoint detection schemes such as SIFT in the overall representation process.

Feature learning-based representation schemes inspired by the BoVW framework have prevailed in the recent past. A dictionary learning-based medical image retrieval method was presented by Srinivas et al. [38]. They proposed to group medical images by sparse representation through existing learned dictionaries using k-singular valued decomposition algorithm. Furthermore, they used orthogonal matching pursuit (OMP) algorithm to match images with dictionaries and associated image clusters. The query image was then compared with images in that cluster to efficiently retrieve relevant images. In a similar approach, Yonggag et al. [39] presented a three stage bag-based framework for medical image retrieval. In the first stage, each image was associated with a relevance degree to a bag (cluster). The stage two, incorporated the image-bag relevance and feature significance to perform pair-wise image similarity. In the final stage, both image-bag similarity and pair-wise image similarities were adaptively combined to retrieve the final results.

Although saliency information is somehow incorporated into the BoVW frameworks, explicit inclusion of visual saliency will improve representational capability. Furthermore, the scalability issues in CBMIR systems when dealing with ever-growing medical multimedia databases has been dealt with cluster-based approaches on visual words frameworks and have shown promising results. Feature learning, inclusion of saliency information in representation, and deriving a compact representation are important factors that will allow efficient and accurate retrieval of medical images.

## 3. Materials and methods

**Fig 1:**
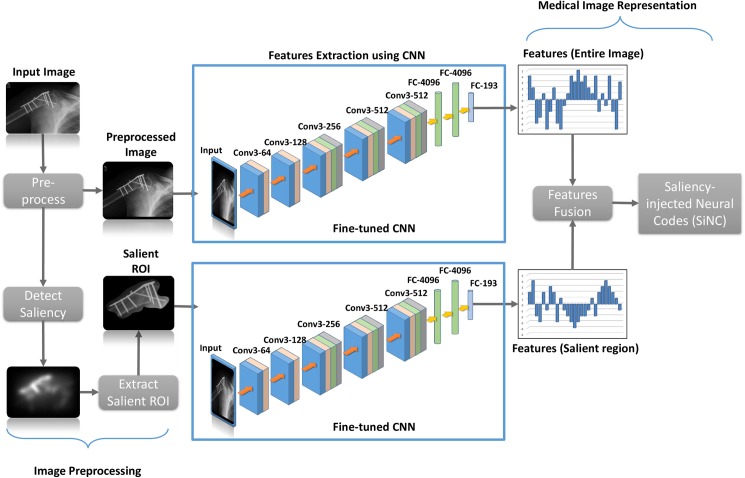
The proposed saliency-injected neural code features for medical image representation.

Incorporating semantic information like visual saliency into image representation frameworks have witnessed improvements in retrieval performance [40, 41]. A closer analysis of radiographs reveal that there exist significant amounts of salient content which can be helpful in improving the retrieval performance if used appropriately. The proposed framework, shown in Fig 1, takes advantage of this semantic information in deriving such a representation that will enable retrieval of radiographs with similarities in the salient contents. It consists of three main components including image preprocessing which involves image rescaling and extraction of the salient image region, features extraction using fine-tuned CNNs, and features fusion. The derived representations are then used to compute image similarities for extracting relevant images to satisfy information needs of radiologists.

### 3.1 Medical image representation

In a CBMIR system, it is imperative to represent images in such a form that visual similarities and mutual semantic relevance can be proficiently determined. Images are usually represented as features corresponding to their visual contents like colors, textures, shapes, and their spatial relationships [42]. CBIR systems rely heavily on image representation schemes. Therefore, most of the effort was usually devoted to feature extraction from images. Features were hand engineered for every CBIR system. However, due to the recent success of deep CNNs in image classification and visual recognition tasks, researchers’ attention has been diverted from feature engineering to automatic feature extraction. This practice allows them to devote their energies in optimizing the overall image retrieval process for particular applications.

Currently, neural activation features are regarded as highly discriminative and robust [43, 44]. These features are extracted by training a deep CNN on a huge image dataset. Millions of parameters (neuron weights and biases) are learned at various layers during the training phase. Each neuron models an input pattern and activates when that particular type of input pattern is encountered. For an image, there exist sets of active neurons in each layer. These activations are regarded as effective representations of the input image which facilitates image recognition and retrieval. Representing medical images using CNNs posed several challenges such as:

1. Difficulty in determining scope and degree of fine-tuning the deep network pre-trained on color images for feature extraction from grayscale medical images
2. It is essential but challenging to automatically model neural activation features to facilitate image relevancy computation according to the perspectives of radiologists.
3. Assessment of features at different layers for image representation
4. Identification and utilization of ROIs according to the radiologists perspectives for medical image modeling

In order to fulfill all these requirements, we propose saliency-injected neural codes (SiNC) framework for representing medical images. Various phases of the whole process are described in the subsequent sections.

### 3.2 Image preprocessing

**Fig 2:**
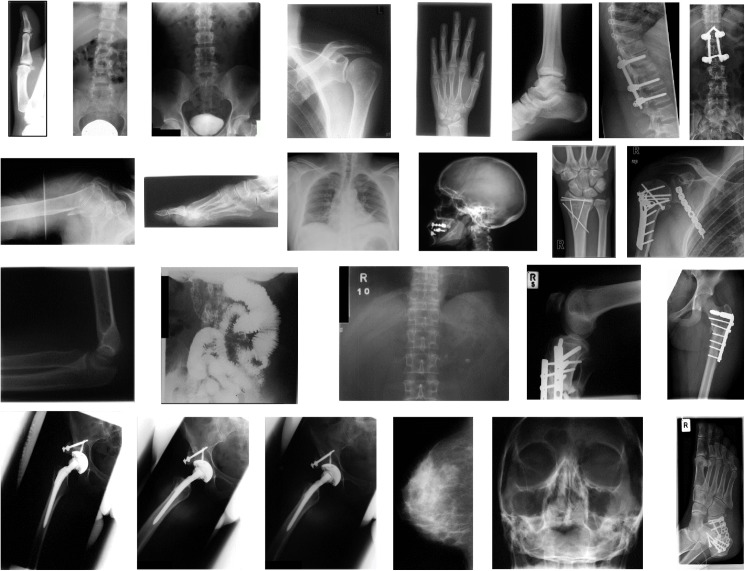
Sample medical images.

The deep CNN we used was trained on color images of fixed size (224 x 224 x 3), thereby requiring inputs to be of the same type and size. In most cases, the input color images are either cropped or rescaled to adjust the size of input for these networks. However, automatic scaling and cropping often removes essential parts of the image or distorts the input which affect the feature extraction process adversely. To extract features from grayscale medical images, they are preprocessed before feed-forwarding through the CNNs. The preprocessing involves, adjusting the input size without distorting the content, and transforming 2D medical images to 3D images for the deep network. The variety in sizes of medical images can be seen in the samples shown in Fig 2. Resizing all images in the same way may affect feature extraction performance. Therefore, an adaptive resizing method, assisted by zero-padding is used to make images compatible for the CNN. For an image *I* having width *w*, and height *h*, the rescaling factor *S*_*f*_ is obtained using the following transformations:

$$S_{f}=\left\{\begin{array}{l} \frac{224}{h},\;h>w\\ \frac{224}{w},\;w\geq h \end{array}\right\}$$ (1)

$$w_{R}=w\times S_{f}$$ (2)

$$h_{R}=h\times S_{f}$$ (3)

where *w*_*R*_ is the rescaled width and *h*_*R*_ is the rescaled height of the image. The rescaled width and height may not be of equal size if the original width and height were not equal. Therefore, the image is padded with zeroes to make it a square image as required by the CNN. If *h* > *w*, then the width of the image is zero-padded such that the image pixels lie in the center of the transformed image. Similarly, the height of the image will be zero-padded, if *w* ≥ *h*. Since, this transformed image is a 2D image, it is transformed to a 3-plane image to fit the input needs of the CNN during fine-tuning. Finally, the mean image values computed over the training images are subtracted from each image in order to center the data around zero. The purpose of this operation is to remove the average brightness from the image because we are more interested in the content of images and not so in their illumination conditions. This preprocessing step has been shown to improve classification performance in the ImageNet dataset [45].

### 3.3 Deep convolutional neural network

Convolutional Neural Networks (CNN) are powerful deep learning architectures applied effectively to solve the problems related to computer vision [44]. Their recent success in large scale image classification (ImageNet) [45], segmentation [46, 47], and face recognition [48] have motivated researchers to utilize these hierarchical networks to learn efficiently about their data. A typical CNN consist of three different neural layers, i.e. convolutional, pooling, and fully connected layers. All of these layers play different roles in the overall modeling process. Network training is accomplished in two stages. Firstly, in the forward stage, the input image is forward propagated through the network with existing parameters (i.e. weights and biases) of neurons at each layer. Then the loss cost is computed using the predicted output and ground truth labels. Secondly, the backward stage calculates gradients of each parameter using chain rules in an attempt to reduce the loss cost. In this way, all the parameters in each layer are updated for the next forward stage. The training stops after sufficient iterations of forward and backward stages or when the loss cost has been sufficiently reduced.

The success of CNN may be attributed to their ability in modeling higher level abstractions in the data. In case of images, the neurons in its various layers become sensitive to particular structures in the visual receptive field. Neurons in the initial layers act like edge detectors and simply react to the various types of edges encountered in images. The inherent hierarchy in deep networks allow neurons in the deeper layers to learn more complex structures. Neuronal activations at the higher layers of these networks contain very useful features which eventually result in higher performance of CNNs in recognition tasks. Razavian et al. [49] and others [50–52] showed that CNNs trained on very large image datasets like ImageNet can act as generic descriptor extractors having powerful discriminative abilities. They exhibited the ability of CNN features in a variety of visual recognition tasks like object classification, scene recognition, and image retrieval etc. achieving very encouraging results.

The successful use of pre-trained CNN features motivated us to use neuronal activations from higher layers of the CNN model trained by Simonyan and Zisserman [53] of the Visual Geometry Group (VGG) of the University of Oxford for representing medical images in our retrieval scheme. The most distinguishing characteristics of this network were its depth and homogeneous convolution and pooling operations across the entire network. The VGG network has 16 layers consisting of 13 convolutional layers and 3 fully connected layers. Each convolution layer consisted of a stack of convolutional layers followed by one pooling layer. The input to the network are fixed size 224 × 224× 3 RGB images preprocessed as described in the previous section. The image is then passed through the stack of convolution layers having small receptive fields of size 3 x 3. In each of these layers, the image is padded to preserve image resolution after convolution. A fixed stride of 1 pixel is used in all the convolutional layers. All the 13 layers in the VGG-16 network are arranged in five stacks. In the first two stacks, there are 2 convolutional layers, and the remaining three stacks had three convolutional layers. Each of these five stacks is followed by a max pooling layer performed within 2 x 2 windows with a fixed stride of 2. The stacks of convolutional layers are followed by three fully connected layers having 4096, 4096, and 1000 neurons, respectively. The final softmax layer outputs predictions for the 1000 classes in the ImageNet dataset.

Though the VGG-16 network was designed for large scale image classification, like other deep CNNs it has also been shown to work as a powerful generic feature extractor [54]. The extended depth and fixed sized convolution and pooling operations allowed for the extraction of useful hierarchical features for efficient visual analysis and recognition of previously unseen content. This characteristic of the network motivated several researchers from the community to utilize these features for a variety of tasks [18, 47, 55, 56].

### 3.4 Fine-tuning

In a recent study, Tajbakhsh et al. [57] showed that CNNs pre-trained on large collections of color and grayscale natural images, when fine-tuned on medical images outperform the CNNs trained from scratch using medical images only. They evaluated both types of CNNs for several medical imaging applications including Polyp detection, Pulmonary Embolism detection, colonoscopy frame classification, and boundary segmentation. They concluded that pre-trained CNNs fine-tuned in a layer-wise manner provide superior performance than other deep learning or hand-crafted representations. The superior performance in all the tasks is attributed to the effective representation of the visual contents obtained through fine-tuning of the model. In a similar study, Gao et al. [58] performed lung disease classification with high accuracy by fine-tuning all layers of a pre-trained CNN. Inspired by their findings, we decided to fine-tune the VGG network to derive an effective representation for medical images. The pre-trained VGG network is designed to recognize 1000 categories of natural objects in the ImageNet dataset. We fine-tuned this network to extract visual features from radiology images. Fine-tuning works on the principles of transfer learning [59], where the classification function of a CNN trained for a broad domain classification problem (e.g. ImageNet classification) is replaced with another classification function (e.g. medical image classification) and optimized to minimize the error in that specific domain. In this way, the features and parameters of the previous network are transferred to the new network with some modification which yield performance improvements. It helps the model transform its focus from a broad, generic domain to a more specific domain.

We replaced the last softmax layer which computes probabilities for the 1000 classes with a new layer which outputs probabilities for the 193 classes in the IRMA 2009 dataset [3]. This new softmax classifier is trained using the backpropagation algorithm on the radiology images. During the fine-tuning process, learning rate for the new softmax layer was kept unchanged as the original rate of 0.01 because it has been randomly initialized. Conversely, the learning rates for rest of the layers were set to 0.001 so that the previous knowledge (parameters) of the network is somewhat preserved and optimized at a relatively gradual pace. The reason behind this strategy is the fact that the VGG network is regarded as an excellent choice for off-the-shelf feature extraction and offers superior performance in a wide range of applications. Furthermore, initial layers of the CNN learn generic low-level image features, which are directly applicable to many computer vision-based applications. On the other hand, the last layers of the network learn application specific high-level features. Therefore, fine-tuning of these layers is usually sufficient. However, since there is a very large difference between the natural and medical images, we opted to fine-tune the entire network. Preserving the parameters while optimizing the network for the medical images will serve the purpose of transfer learning at best. The network was optimized using stochastic gradient descent (SGD) in 34K iterations using NVidia DIGITS system [60]. A batch size of 20 was used and the fine-tuning process was executed for 10 epochs.

After the fine tuning, neural activation features were extracted from the last fully connected layer of the network for representing medical images. The basic concept of image representation is to compute a set of feature values *f* = {*f*_1_,*f*_2_,…,*f*_*n*_} from image *I*, such that feature similarity can be translated into image similarity. Neural codes ($N_{C}^{I}$) were obtained from the fine-tuned VGG network:

$$N_{C}^{I}=ExtractFeatures(I,VGG\_FT,Layer)=\{f_{1},f_{2},\ldots ,f_{n}\}$$ (4)

where $N_{C}^{I}$ represent the neural codes for image *I*, *ExtractFeatures()* is a function which extracts features from the specified layer (i.e. *Layer* = FC8) of the fine-tuned model (i.e. VGG_FT) by feed-forwarding input image *I*, and *f*_*i*_ refers to the activation value of the *i*^th^ neuron in the specified layer.

### 3.5 Salient content in medical images

**Fig 3:**
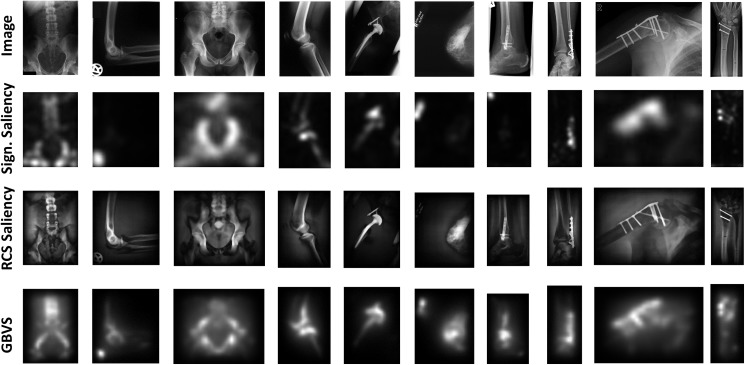
Saliency detection in medical images.

Medical image retrieval aims at identifying and searching images with similarities according to the interests of radiologists. Such similarity refers to the presence of particular peculiarities in images such as tumors, calcified spots, and fractures, etc. These patches of images are often the regions of interest to the medical expert who intends to search and analyze previous relevant medical cases prior to making clinical decisions. To identify these regions of interest in medical images, we propose to employ saliency detection methods for localizing such peculiarities in images prior to feature extraction. This additional information induced into the feature extraction process will result in more accurate identification of relevant contents in CBMIR applications. Furthermore, the saliency models in general are able to identify significant regions in medical images which will help in retrieval performance improvement even in the absence of such peculiarities. Results of some famous saliency detection schemes including graph-based visual saliency (GBVS) [61], signature saliency [62], and random center surround saliency (RCS) [63] on a variety of medical images is given in Fig 3. It can be seen from the results that the saliency detection methods are able to identify regions of interest. Specifically, GBVS method has successfully highlighted the salient image patches in most of the medical images which makes it appropriate for saliency detection in these images. It is a bottom-up saliency model which is based on graph computations. For an image *I*, the objective is to highlight significant portions of the image based on a criterion such as human fixation data density. For a given feature map *M*, an activation map *A* is computed such that pixel location (*i*, *j*) in *A* will contain high activation values if the *M(i*,*j)* is unusual from its surroundings. For computing the local dissimilarity between two distinct locations *M(i*,*j)* and *M(p*, *q)* in the feature map, a logarithmic dissimilarity metric *d* was employed such that:

$$d((i,j)\|(p,q))=\left|\log\frac{M(i,j)}{M(p,q)}\right|$$ (5)

Every node (i.e. pixel) in the feature map *M* is connected with the remaining *n*-1 nodes in *M* to form a fully connected directed graph *G*_*A*_. The node *(i*, *j)* has a directed edge towards *(p*, *q)* having weights

$$w_{1}((i,j),(p,q))=d((i,j)\|(p,q)).F(i-p,j-q)$$ (6)

where

$$F(a,b)=\exp\left(-\frac{a^{2}+b^{2}}{2\sigma^{2}}\right)$$ (7)

The parameter *σ* in (7) was set to one tenth or one fifth of the width of *M*. It is evident that the weight *w* of the directed edge is proportional to the dissimilarity and closeness in *M*. A Markov chain define on *G*_*A*_, whose equilibrium distribution helps accumulate mass at nodes having high dissimilarity with neighboring nodes. This yields an activation measure derived from pair-wise contrast. Like the leading saliency computation schemes which highlight very limited areas of images, GBVS method concentrates mass on activation maps in order to make the saliency map more informative. The final saliency map *S* computed by treating *G*_*A*_ as a Markov chain followed by computation of the equilibrium distribution over the nodes of the graph. Since GBVS is a mass concentration algorithm, mass will flow towards the nodes with high activations. Saliency detection methods, especially GBVS performed substantially well in identifying regions of interest automatically. The identified salient patches were extracted from the image using (8), rescaled according to the procedure explained in Section 3.2. Afterwards the salient patch is feed forwarded through the network to acquire neural codes for the salient part *I*_*S*_.

$$I_{S}(x,y)=\left\{\begin{array}{l} I(x,y),\;S(x,y)\geq \tau \\ 0,\;Otherwise \end{array}\right\}$$ (8)

$$\tau =\frac{1}{MN}\sum_{x=1}^{M}\sum_{y=1}^{N}S(x,y)$$ (9)

where *S(x*,*y)* is the value in saliency map at location *(x*,*y)*, *τ* is the threshold value computed as the mean value of the saliency map *S*, *M* and *N* are the width and height of the *S*, respectively. It allows dynamic selection of the threshold based on the saliency map. *I*_*S*_(*x*,*y*) contains the pixel value at position *(x*,*y)* in *I* which belongs to the salient part of the image determined by τ.

### 3.6 Saliency-injected neural codes

Final features are computed by fusing neural codes of the entire image $N_{C}^{I}$ and salient image patch $N_{C}^{S}$. This fusion process acts as a means to inject saliency information into the neural activation features to build a stronger discriminative representation. The fusion process is performed as:

$$SiNC=\alpha N_{C}^{I}+\beta N_{C}^{S}$$ (10)

where *α* and *β* are relative weights assigned to the two neural codes such that *α* + *β* = 1, and $N_{C}^{S}$ are the neural codes for the salient part of the image computed as:

$$N_{C}^{S}=ExtractFeatures(I_{S},VGG\_FT,Layer)=\{f_{1}^{S},f_{2}^{S},\ldots ,f_{n}^{S}\}$$ (11)

This weighted scheme provides a degree of freedom to adjust the system parameters to best fit the current query scenario. For majority of the queries, we used the values of *α* and *β* to be 0.4 and 0.6, respectively. It can be adjusted according to the requirements of the query being processed.

### 3.7 Short binary codes for large scale image retrieval

Locality sensitive hashing (LSH) methods provide efficient means to retrieve images from huge image collections by reducing the search space significantly. These methods tend to solve the nearest neighbor search problems, where the objective is to quickly and accurately locate visually similar nearest neighbors in a database of images (*I*_*1*_, *I*_*2*_,..*I*_*N*_) to a query image *I*_*Q*_. LSH methods work on the principle of approximate similarity search where retrieval performance is sacrificed for allowing fast queries. The basic concept is to project the high dimensional feature vectors to a low-dimensional hamming (binary) space such that each feature vector is mapped to a b-bit vector known as the hash key. The value of *b* determines the number of bits to be used for representing a feature vector. The hash key can be used to effectively locate nearest neighbors of the query image in sub-linear time, given that the projection is performed appropriately. For indexing the images using this scheme, a hash table is constructed by applying *b* binary valued hash functions (*h*_*1*_, *h*_*2*_, *… h*_*b*_) to all database images. Each of these hash functions must satisfy the locality sensitivity criteria:

$$\mathrm{Pr}[h(I_{i})=h(I_{j})]=sim(I_{i},I_{j})$$ (12)

Where *Pr* is the probability that the hash keys for two images *I*_*i*_ and *I*_*j*_ will be similar and *sim*(*I*_*i*_,*I*_*j*_) ∈ [0,1] is the similarity function used for pair-wise image matching. It means that visually similar images will have high probability of collision in the hash table (i.e. the same hash key will be assigned to visually similar images). Given the query image *I*_*Q*,_ its hash key is computed using the same *b* hash functions. The hash key collides with a certain bucket in the hash table, which points to a small portion of stored samples/images. Only those images are retrieved without exhaustively searching the entire dataset. This scheme allows retrieval of (1+ε)-near neighbors in O(n^1/(1+ε)^) [64].

Several approaches have been presented in the past to generate hash codes including locality sensitive hashing (LSH) [65], Kernelized locality sensitive hashing (KLSH) [64], Spectral Hashing (SH) [66] and Iterative Quantization (ITQ) [67], etc.

In our case, we applied KLSH scheme on the SiNC feature vector to obtain a compact representation of *b* bits. The SiNC feature vector was normalized prior to applying the KLSH algorithm using:

$$SiNC_{}^{N}=\frac{SiNC-m_{n}}{m_{x}-m_{n}}$$ (13)

where *m*_*x*_ and *m*_*n*_ are the minimum and maximum activation values of the SiNC descriptor for all images in the dataset and *SiNC*^*N*^ is the normalized feature vector.

The hash keys are constructed by applying *b* binary valued functions (*h*_1_,*h*_2_,…,*h*_*b*_) to *SiNC*^*N*^ such that the locality sensitive hashing property is satisfied:

$$\mathrm{Pr}[h(SiNC_{i}^{N})=h(SiNC_{j}^{N})]=sim(SiNC_{i}^{N},SiNC_{j}^{N})$$ (14)

where $sim(SiNC_{i}^{N},SiNC_{j}^{N})$ is the similarity function for computing image similarity between candidate images having values between [0, 1]. It is desired to have collisions in the hash table with similar examples, so that nearby locations in the hash table will contain visually similar medical images. In KLSH, Kulis and Grauman kernelized the input data by applying radial basis function (RBF) kernel on the input vector *SiNC*^*N*^ = (*x*_1_,*x*_2_,…,*x*_*n*_) to obtain *ϕ*(*x*) such that the input data can only be accessed through this kernel function $K(SiNC_{i}^{N},SiNC_{j}^{N})=\phi {\left(SiNC_{i}^{N}\right)}^{T}\phi (SiNC_{j}^{N})$. A subset of ρ samples (SiNC feature vectors) are chosen to define the RBF kernel matrix K over these sampled data points. The kernel matrix is zero-centered, and a hash table is constructed by selecting *t* indices randomly from [1,… *ρ*] to form ***e****s*. The final values for all the *b* bits are computed by determining the sign from the hash functions:

$$h(\phi (x))=sign(\sum_{i}w(i)\kappa (x,x_{i}))$$ (15)

where *w*(*i*) is the *i*^th^ value in weight matrix for computing hash keys over the queries and is calculated as *w* = *K*^−1/2^***e****s*, ***e*** is a vector of all ones, and ***e****s* is a vector with ones in the corresponding indices of Z, which is the set of selected database items for computing kernel matrix K. Consequently, a compact *b*-bit binary representation is constructed for each image in the dataset as well as the query image to accomplish efficient retrieval in large scale datasets, where the relevant images are located by employing existing nearest neighbor search (NNS) algorithms.

## 4. Experiments and results

This section presents the evaluation dataset, experimental setup, implications of various parameters on retrieval performance, and discussions on various experiments.

### 4.1 Image database

To assess performance of the proposed representation and retrieval framework, we used a database provided by IRMA group from the University Hospital of Aachen, Germany [3]. It contains diverse sets of radiology images of hand, skull, chest, knee, shoulder, neck, breast, foot, etc. It consists of 15363 images in total, in which 13630 are provided for training and the remaining 1733 are used for testing purposes. All the images were annotated and categorized into 193 distinct groups, where each group contain a number of semantically relevant images. The test images were used as queries to extract relevant images from the training set. Maximum image dimension in this dataset is 512 and minimum is 120.

### 4.2 Experimental setup

The proposed system was implemented in MATLAB 2015a for Windows 7 Professional running on a PC equipped with 8 GB RAM, 3.4 GHz Intel Core i5 processor, and NVidia GeForce GTX 650 GPU. Network fine-tuning was performed on an Intel Core i7 processor equipped with 16 GB memory and NVidia GeForce GTX TITAN X with 12 GB of onboard memory, running NVidia DIGITS 3.0 [60] system in Ubuntu 15.10. For fine-tuning the network, 13630 images were randomly cropped into 224 x 224 sub images to generate 68000 training images. The remaining 1733 images were used as queries for retrieving relevant images. Different sets of experiments were designed to assess the overall performance of the proposed scheme.

### 4.3 Evaluation metrics

Performance of image retrieval systems are usually measured in terms of precision and recall. Precision shows the capability of the technique to retrieve relevant images. Recall exhibits the portion of relevant images retrieved from all the relevant images in the dataset. An ideal system will achieve high precision values for all recall settings. However, it is usual observation that precision drops at high recalls. The objective is to maintain a high precision value for high recall rates. These values are measured as:

$$P=\frac{N_{R}}{N_{R}+N_{I}}$$ (16)

$$R=\frac{N_{R}}{T_{R}}$$ (17)

where *N*_*R*_ is the number of relevant images retrieved, *N*_*I*_ is the number of irrelevant images retrieved, and *T*_*R*_ is the total number of relevant images in the database. Based on these two metrics, a precision-recall curve is usually plotted to show the retrieval performance of an algorithm for various recall settings. Furthermore, a unified performance metric derived by computing area under the precision-recall curve (AUC) is also computed for the proposed method and compared with several existing methods.

### 4.4 Evaluating feature layers

**Fig 4:**
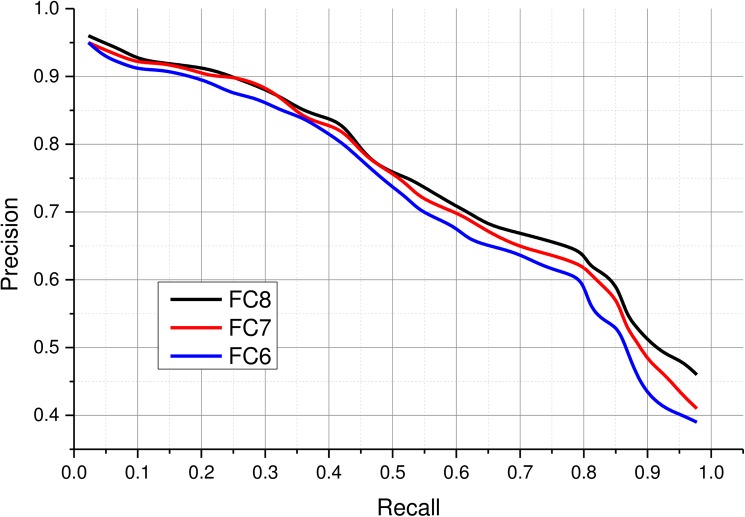
Retrieval performance of the proposed CBMIR scheme with neural codes extracted from various fully connected layers.

A CNN typically learns different layers of features from images during the training process. Neuronal activations from the fully connected layers usually provide effective representations of the input which are frequently used to perform visual recognition tasks [68]. In this experiment, we evaluated performance of features obtained from the last three fully connected layers of the fine-tuned model. Several experiments were performed to assess the suitability of these layers for representing medical images. Although, features from all the fully connected layers possessed sufficient discriminatory capabilities to perform image retrieval, it is necessary to determine the best set of features. After evaluating features extracted from different layers for various queries, it was found that the last fully connected layer FC8 performed slightly better than the previous two layers due to the fact that higher layers abstracts higher level semantic concepts than the lower layers. Furthermore, this layer is much more compact (193 neurons) than the previous two layers having 4096 neurons and it also contains the most comprehensive features for performing the desired retrieval task. Precision values for various recall settings are reported in Fig 4. All the layers maintain a high precision (>0.9) for recall settings below 0.2. The overall precision drops gracefully for all the layers as the number of retrieved images is increased. A slight improvement can be seen in the top 10% for FC8 over other layers. Beyond recall 0.3 up to 0.7, a gradual degradation is observed in retrieval performance for all the layers, with FC8 performing slightly better. However, for recall beyond 0.65, FC8 maintains a significantly better precision as compared to FC7 and FC6. At recall settings beyond 0.8, the precision of all the layers drop significantly especially FC6 suffers the highest performance loss. In terms of area under the precision recall curve (AUC), FC8 achieves 0.73 AUC compared to 0.72 and 0.69 for FC7 and FC6 respectively. High performance and short feature length of the FC8 motivated us to use this feature for the proposed CBMIR system.

### 4.5 Retrieval performance of SiNC

**Fig 5:**
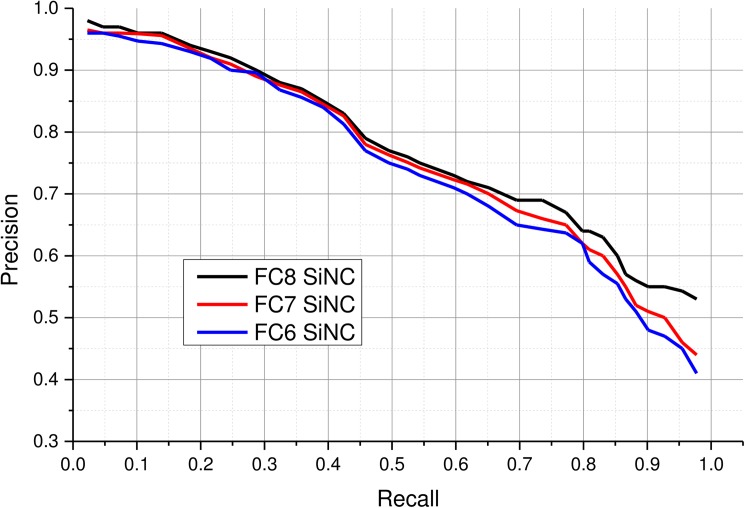
Retrieval performance of the proposed CBMIR scheme with SiNC.

Neural codes from the pre-trained CNN performed substantially well for grayscale medical images and were able to successfully retrieve relevant images in most of the queries. However, their retrieval performance in images with peculiarities was not as good as their performance in retrieving normal images. In this context, we experimented with inclusion of saliency information and fine-tuning to proliferate discriminative capability of the SiNC descriptor in order to improve its overall retrieval performance as reported in Fig 5. With the inclusion of saliency information, retrieval performance for all the layers has been improved as is evident from the precision recall curve. Similar to the results in Fig 4, FC8-SiNC maintains slight edge over other layers while retrieving with recall up to 0.3. Precision scores drop in the same way for all the layers up till recall 0.7. Beyond this point, FC8-SiNC maintains better scores for all subsequent recall settings. FC8-SiNC achieves 0.75 AUC which is better than FC6-SiNC and FC7-SiNC by 3.6% and 1.9%, respectively.

**Fig 6:**
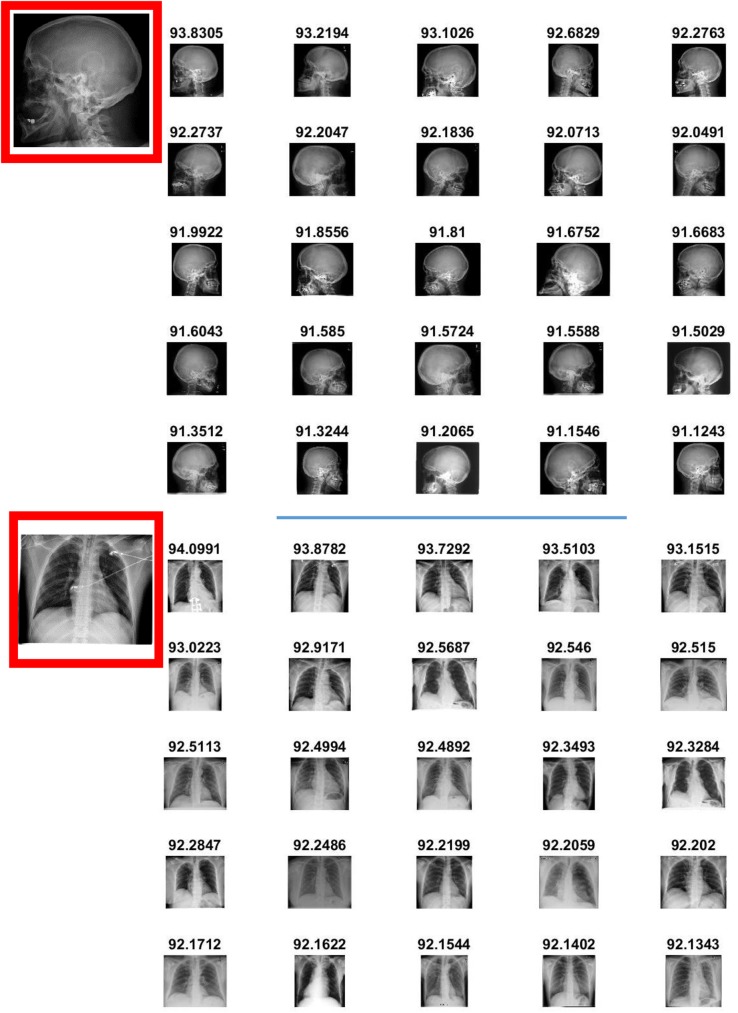
Retrieval results for medical images without medical peculiarities.

**Fig 7:**
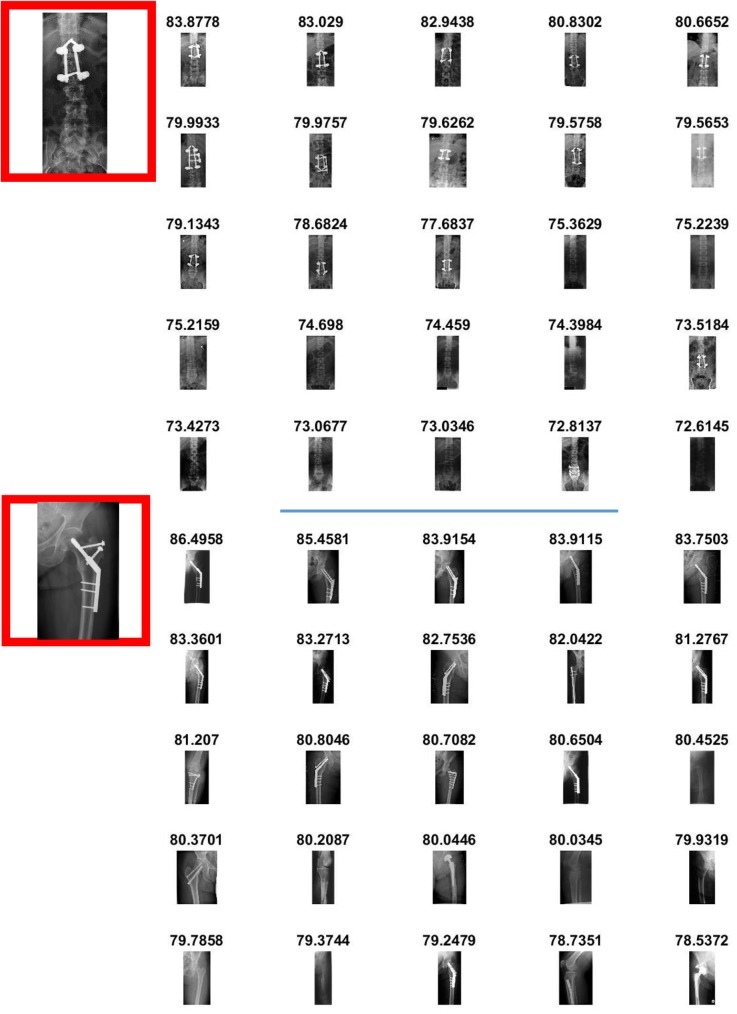
Retrieval results of medical images with different peculiarities.

The effectiveness of SiNC features for medical image retrieval can also be seen from the visual retrieval results for different queries. Fig 6 show retrieval results for two different query images enclosed within red boxes. A total of 25 images were retrieved for each query image taken from the set of query images and relevant images were retrieved from the database. The scores written on top of each retrieved image represent the similarity score. It can be noticed that the similarity scores for identical images are very high for visually similar images. An interesting point to note here is that horizontally flipped images are also retrieved by the system which exhibit the invariance capability of the SiNC descriptor. In Fig 7, the query images contain joint support rods and therefore radiologists would be interested in both visual similarity and salient content uniqueness as the query image. Since the proposed SiNC descriptor embeds visual saliency features in the representation process, semantically relevant images were retrieved for both queries at top ranks which exhibit its suitability for retrieval of such challenging images.

**Fig 8:**
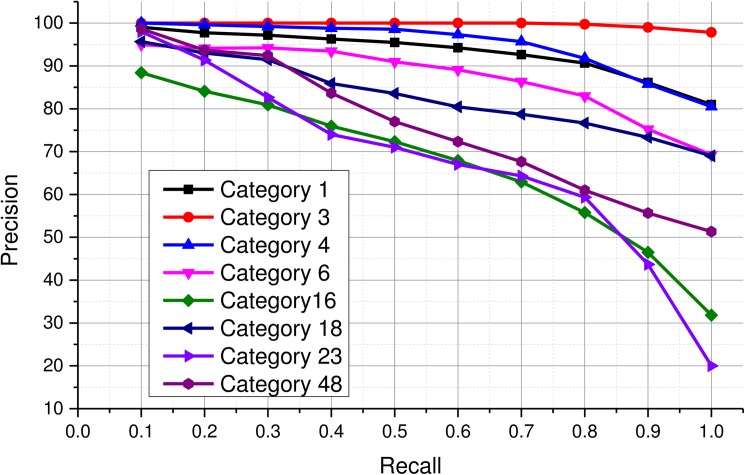
Category-wise retrieval performance using SiNC.

In order to assess the retrieval performance of SiNC for various categories in the IRMA 2009 dataset, precision-recall values are reported for some major categories in Fig 8. It can be observed that images from majority of the categories were retrieved with more than 95% precision at recall 0.2. At recall setting 0.3, precision above 90% is achieved for six of these categories. All of the relevant images belonging to categories 1, 3, 4, 6, and 18 were retrieved with more than 70% precision for all recall settings. However, the precisions for other categories 16, 23, and 48 dropped significantly beyond recall 0.8. The reason behind this significant decrease in the retrieval performance for these categories was that some of the images in these categories had significant illumination variations and occlusions which lead those images be confused with images from other categories. Therefore, precision values dropped significantly when those images were retrieved from the dataset.

Table 1 shows the comparison of different image representation schemes for radiographs obtained from a freshly trained CNN with the same architecture as VGG16, pre-trained VGG model, fine-tuned model, and SiNC. From the results reported in the table, it can be seen that SiNC descriptor performs better in comparison with other representations. It performs better than VGG16 raw features by 9% at 0.1 recall and 32% at recall 1.0. Similarly, it outperforms fine-tuned VGG features-based representation by almost 3%, and concatenation scheme of fine-tuned VGG features and saliency features by 2% at 0.1 recall. For recall settings 1.0, SiNC descriptor outperformed fine-tuned VGG16 features by more than 12%. It is interesting to note here that the fine-tuning process lead to improved retrieval performance despite the fact that the VGG16 model was pre-trained on a very different set of natural color images.

**Table 1:** Average precision scores at various recall settings for different representation schemes.

	Recall				
Representation Method	**0.1**	**0.25**	**0.5**	**0.75**	**1.0**
Newly Trained VGG16	0.81	0.72	0.60	0.46	0.27
VGG16_FC8	0.87	0.83	0.69	0.51	0.36
VGG16_FT_FC8	0.93	0.90	0.76	0.65	0.47
VGG16_FT_FC8+Saliency	0.94	0.90	0.77	0.66	0.49
SiNC	0.96	0.92	0.77	0.68	0.53

**Fig 9:**
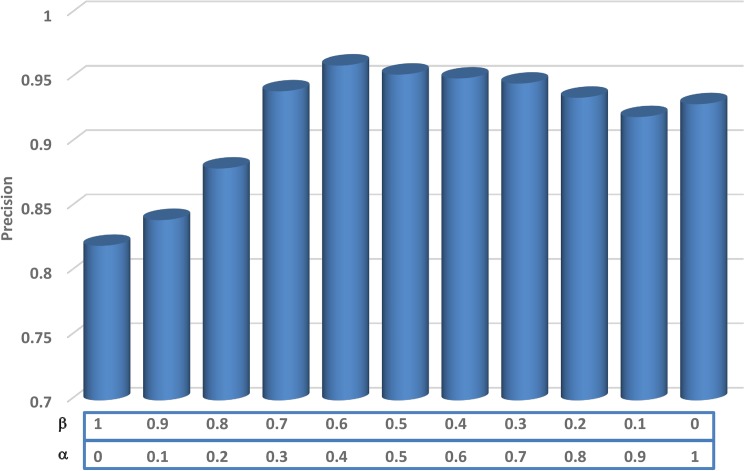
Effect of saliency injection on retrieval performance.

Images having sufficient visual information about the anatomy like radiographs of chest (category 1, 2, 3, 25, 52), abdomen (categories 6, 7, 14), skull (categories 4, 10, 12, 18), shoulder (29, 37, 45, 48, 78), hand and feet (5, 28, 31, 34, 81, 82, 99), knee (24, 26, 30), and neck (9) were retrieved with high precision for recall ranges 0.1 to 0.7. On the contrary, the proposed method has slight difficulty with images of fingers, and parts of arm and leg bones at high recall settings due to the fact that some of the images in these categories could be easily confused with other visually similar images. Retrieval results for some challenging queries are provided in Fig 9. Images enclosed within the red rectangles are the query images. In the top row in each query, are the top-10 retrieved images by the SiNC descriptor. The second row contains top-10 images retrieved by the pre-trained VGG16 network features. It can be seen in all of these queries that SiNC is able to retrieve more relevant images than the VGG16 features. In the first query, the proposed method retrieved all the images correctly, whereas only three relevant images are retrieved by the VGG16 features. Similarly, in the rest of the queries, more visually similar and relevant images are retrieved by SiNC with significantly higher precision than the other scheme.

### 4.6 Effect of saliency injection on retrieval performance

Recent advances in visual saliency detection has enabled computer vision systems of simulate this essential aspect of human visual system in identifying the most significant object in image. Once these regions are identified, features pertaining to these regions are usually given higher weights and significance than the rest of the image. Such approaches in image retrieval have shown promising results [12, 69, 70]. In this study, we chose to incorporate visual saliency for identifying regions of significant importance to the radiologists. Experiments have revealed that the chosen saliency detection scheme is capable of identifying fractures, calcified spots, and tumor regions in images. In this section, we provide a thorough analysis of inclusion of visual saliency into the image description process, and study the effects of saliency injection on retrieval performance.

In the proposed framework, the role of salient features in image representation is controlled by two parameters *α* and *β*. By adjusting their values, we can control the inclusion of saliency into the image representation. During these experiments, we evaluated different values for these variables and studied their effects on retrieval performance and determined an optimal set of values for these essential parameters. Fig 9 shows the effects of saliency injection on retrieval performance for top 10% retrieved images (precision at 0.1 recall). It can be seen that modifying the values of *α* and *β* enables us to control the degree of saliency features to be included in the image representation process. By setting the value of *α* to 0 and *β* to 1, results in a low precision score of 0.78 which is due to the fact that only features from the salient region are used to retrieve images without the contextual information. By improving the value of *α*, significant improvements were recorded due to the inclusion of contextual features. Similarly, we can ignore the saliency features and use only the deep features of the entire image by setting the value of *α* to 1 and *β* to 0. At this setting, we achieved a precision of 0.93. Through these experiments, we determined optimal values for both *α* and *β* to be 0.4 and 0.6, respectively.

**Fig 10:**
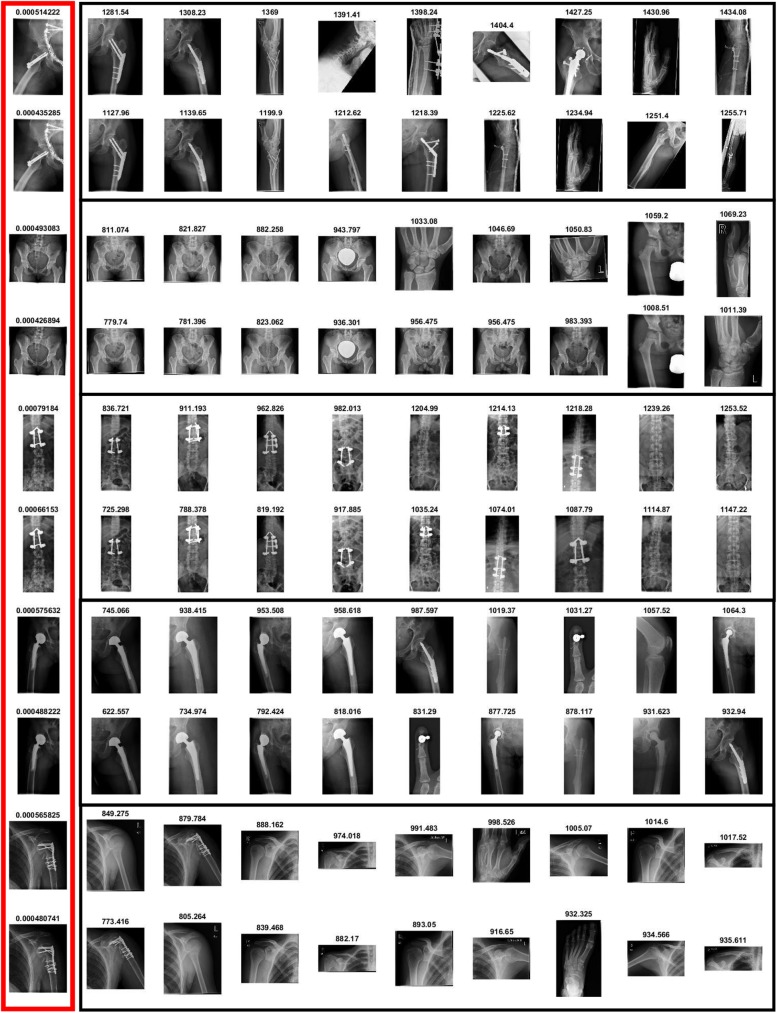
Top retrieved images using deep features (first row) and SiNC features (second row).

Qualitative results for some queries have been provided in Fig 10. Some of the images have salient regions like fractures, and calcified spots, whereas other do not have any significant salient regions. Still, the proposed method yielded superior performance as compared to simple deep features. The query image is shown to the left, the retrieved images using simple deep features are provided in the top row of each query, whereas the retrieval results using the proposed approach are given in the second row. It can be seen that for almost all the queries, the proposed SiNC features yielded better results. In the first, third, fourth, and last query, there exist fractures in query images, the SiNC features retrieved more relevant images at higher ranks than the simple deep features. Similarly, it provided superior retrieval performance even if there exist no such salient region as exhibited in second query.

### 4.7 Sensitivity analysis of proposed framework

**Fig 11:**
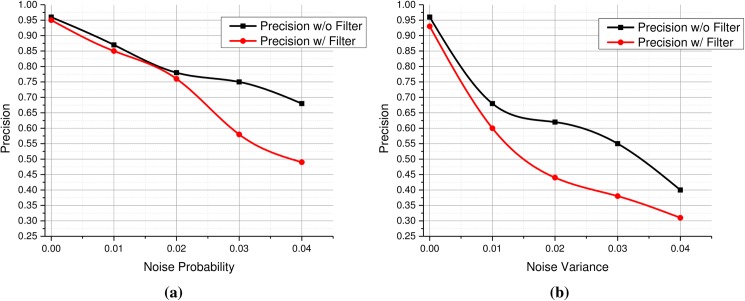
(a) Precision scores with varying probabilities of Salt & Pepper noise (with and without filtering), (b) Precision scores with different variances of Gaussian noise (with and without filtering).

The VGG model used for features extraction is a key component of the proposed framework and its optimal utilization is highly essential for the success of our system. For this purpose, we carried out sensitivity analysis of the model in order to make sure that we get the best out of this model for image retrieval purposes. To a certain degree, we have already performed sensitivity analysis of the VGG model. For instance, we studied the comparative performance of various layers of the model to determine the best set of features for medical images. Similarly, we also carried out an empirical evaluation of the parameters *α* and *β* to select optimal values for features fusion. Since we used the fine-tuned model, we could not modify its overall architectures to a great extent. We only could modify the last FC layer according to the number of classes in our dataset and kept the rest of the architecture unchanged in order to utilize the previous parameters as much as possible. However, it is essential to study the robustness of the model to several image degradations and transformations which the proposed system may come across in practical environments. In radiographic images, two important challenges are noise and random translations. So we studied the effects of these image degradations and transformations. For this purpose, we conducted several experiments to assess the effects of various kinds of noises and the precision for Salt & Pepper noise have been shown in Fig 11(A) and 11(B) highlights the results of Gaussian noise with different variance. Gaussian noise caused higher performance degradation as compared to Salt & Pepper noise. In both cases, filtering attempts to reduce noise caused significant degradation in retrieval performance. Hence we recommend to avoid filtering the query image before features extraction. We also evaluated the robustness of the model to translations and image cropping, and found that the VGG model is very robust to these transformation, partly due to its robust nature and partly due to the fact that we used random crops of images to fine-tune the model. Experimental results revealed that random translations and cropping do not affect the retrieval performance of the proposed system, if major portion of the image remains intact.

### 4.8 Feature hashing for scalable image retrieval

**Fig 12:**
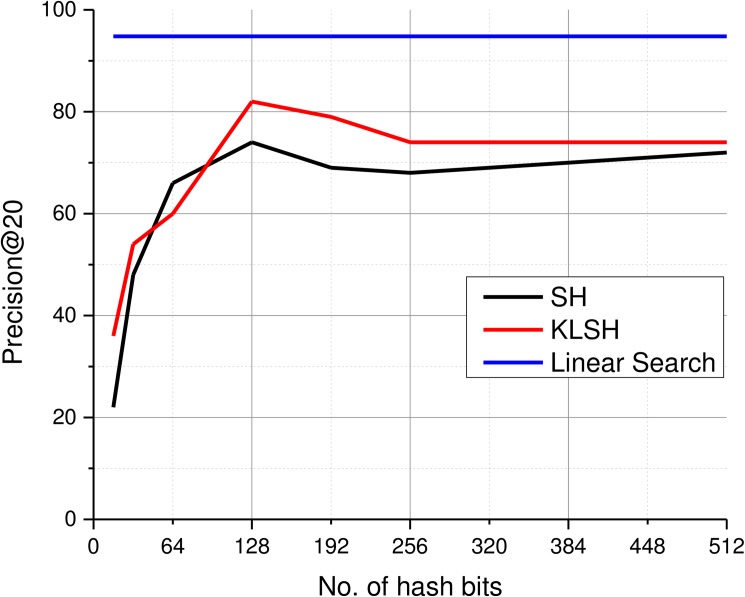
Retrieval performance with different hash code lengths.

**Fig 13:**
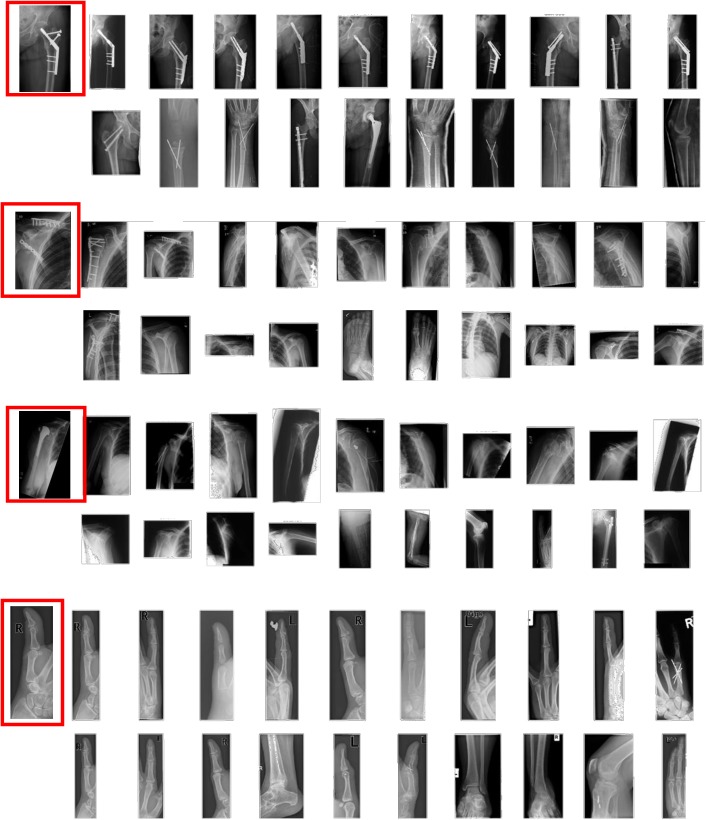
Retrieval performance with different representation schemes for some queries. Top row of each query represent retrieval using SiNC features and the second row shows retrieved results using simple deep features.

**Fig 14:**
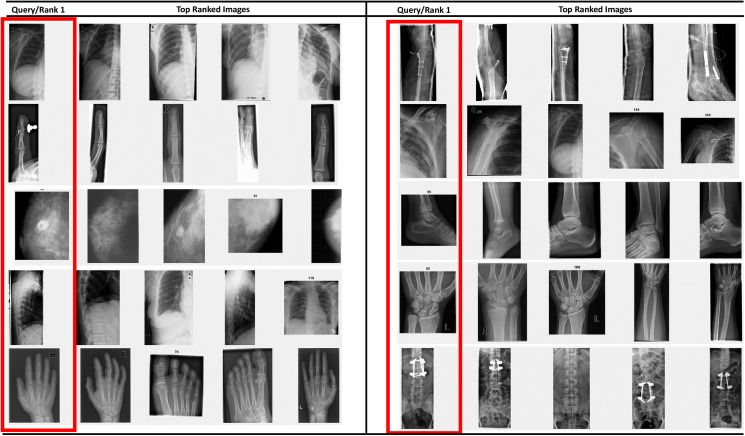
Retrieval results with 128-bit hash codes of SiNC (The first column contains the query images (top-1 ranked image) and the remaining columns contain the top-4 ranked images).

Large scale image retrieval demands efficient access to relevant information within huge image collections. Linearly searching the entire database becomes infeasible for very large and constantly growing databases like medical images. To address this issue, researchers have proposed several methods for computing hash keys to enable faster query execution at the cost of performance reduction [23, 24]. For the proposed SiNC features, we evaluated two different hashing schemes and concluded that the existing hashing methods like Kernelized LSH and Spectral Hashing can be used to build hash tables for allowing faster queries in large image databases. These methods can be used to draw an initial set of images using NNS algorithms and then the search can be refined by performing linear search in that subset using the SiNC features. We performed several experiments with different hash key lengths for both KLSH and SH and found that both methods perform the best with 128-bit hash codes. Fig 12 reports the outcomes of our experiments. For prec@20, KLSH method achieved more than 80% retrieval accuracy. Fig 13 reports retrieval results using hash features obtained using SiNC and VGG deep features. Results reveal that the proposed SiNC features yielded better retrieval performance. Fig 14 shows results of ten different queries executed using KLSH with hash keys of 128 bits. It can be seen that the top-5 retrieved results mostly contain relevant images.

Comparison of the query execution time (in milliseconds) with and without hashing is provided in Table 2. For each case with varying database sizes, 300 random queries were run and average retrieval times along with standard deviations have been reported. Retrieval with hash codes is 2.6 times efficient than querying without hash codes in a database of 15K images. For a significantly larger dataset, the improvement of SiNC + KLSH is much more pronounced. Increasing the search space increases the amount of retrieval time for linear search. On the other hand, the retrieval time for hashing-based approach remains almost constant. All these values are computed for MATLAB-based implementation.

**Table 2:** Retrieval efficiency with and without hash codes.

Database Size (Number of images)	Average Retrieval Time (ms) (SiNC + Linear Search)	Average Retrieval Time (ms) (SiNC + KLSH)
2,000	80 ± 4.1	127 ±3.7
5,000	191 ±6.3	130 ±3.3
15,000	346 ±6.6	137 ±4.0
60,000	1221 ±17.7	141 ±3.3

### 4.9 Comparative analysis with other CBMIR methods

**Fig 15:**
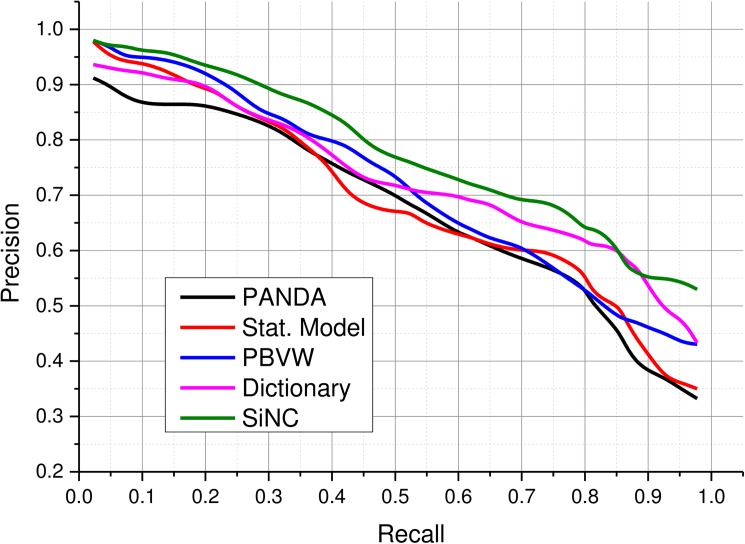
Performance comparison with other CBMIR approaches.

This section presents a comparison of SiNC with several existing schemes on the IRMA dataset and provides critical analysis on several performance aspects. The PANDA framework developed by Iakovidis et al. [33] used pattern classes to keep track of similar patterns in medical images which assisted in determining structural similarities between image pairs. Since they used low-level features to describe local patterns, their method offered comparatively lower precision at recall settings below 0.3 as depicted in Fig 15. Furthermore, they did not consider the interest of medical experts while retrieving images from the dataset. Their method achieved 66.75 AUC and its performance dropped gradually when more and more images were retrieved and a significant drop was observed beyond 0.8 recall. In Stat. Model framework [25], the use of low-level features also affected retrieval performance, partly due to the absence of color in medical images and partly due to the weak representational strength of these features. Their method achieved 67.20 AUC, however, their performance dropped significantly as a result of increasing the number of retrieved images. The method in [35] used the BoVW framework to represent densely sampled image patches. Their system was able to discriminate between healthy and pathological cases and therefore, achieved better retrieval performance than the previously discussed methods. Srinivas et al. [38] method used sparse coding features to identify dictionaries and associated image clusters to retrieve relevant images from the dataset. Their method achieved retrieval accuracy above 90% at 0.2 recall which dropped to 43% at full recall. Like most of the existing methods, they did not consider region of interest while retrieving images. Therefore, it failed to deal with queries involving a very particular region of interest. The proposed method achieved steady precision for recall up to 0.85. This improved performance verifies the representational capability of SiNC for retrieving medical radiographs.

Table 3 lists AUC values of the proposed method and other similar methods. The proposed scheme provides superior performance in terms of precision and recall than several existing methods on the evaluation dataset. Similarly, the hashing-based scheme also provides reasonable performance (82% for P@20) slightly below the linear search scheme. The proposed method is believed to provide promising performance for large scale datasets. We also believe that the inclusion of cues for specifying ROIs for visual content queries like image saliency has great potential for retrieval applications and needs to be studied further.

**Table 3:** Comparison of area under the precision recall curve for IRMA 2009 dataset.

Performance	Methods				
	PANDA [33]	Stat. Model [25]	PBVW [35]	Dictionary [38]	SiNC
AUC	66.75	67.20	70.61	71.0	75.17

## 5. Conclusion

In this paper, an efficient method for representation and retrieval of medical images is presented. Our objective is to enable efficient retrieval of medical images having visual and semantic similarities. Saliency detection method was used to automatically identify regions of interest in medical images. These methods were able to highlight regions containing fractures, calcified spots, and tumors. Neuronal activations from the fully connected layers of a fine-tuned CNN were studied for the suitability of representing medical images. A deep CNN trained by the visual geometry group (VGG) was used in this study. It was found that the last fully connected layer represented images more efficiently than the other two fully connected layers. Neural codes were extracted from the entire medical image as well as the salient part. Fusion of both these features yield the final feature vector used to index medical images. Experiments conducted with the IRMA dataset revealed that the proposed SiNC descriptor is sufficiently discriminative for retrieving relevant medical images from large image collections. The inclusion of saliency information in the feature extraction process helped in identifying semantically relevant images to better fulfill the needs of radiologists and other MEs. For most of the queries, we were able to retrieve relevant images, however, in certain cases where the saliency detection method failed to identify the region of interest appropriately, relevant images were scarcely retrieved in the top ranked results. Further study is needed to make the existing framework more efficient by visual saliency detection method for identifying medical peculiarities, and directly learning binary hash codes from CNNs to facilitate retrieval from large scale databases. Moreover, iterative query refinement and relevance feedback schemes can be incorporated to further improve retrieval performance of our framework.
